# Coordinate and synergistic effects of extensive treadmill exercise and ovariectomy on articular cartilage degeneration

**DOI:** 10.1186/s12891-016-1094-8

**Published:** 2016-05-31

**Authors:** Kazumasa Miyatake, Takeshi Muneta, Miyoko Ojima, Jun Yamada, Yu Matsukura, Kahaer Abula, Ichiro Sekiya, Kunikazu Tsuji

**Affiliations:** Department of Joint Surgery and Sports Medicine, Tokyo Medical and Dental University, Tokyo, 113-8519 Japan; Center for Stem Cell and Regenerative Medicine, Tokyo Medical and Dental University, Tokyo, 113-8519 Japan; Department of Cartilage Regeneration, Tokyo Medical and Dental University, 1-5-45 Yushima, Bunkyo-ku, Tokyo 113-8519 Japan

**Keywords:** Ovariectomy, Mechanical stress, Forced running, Joint inflammation, Naturally occurring osteoarthritis model

## Abstract

**Background:**

Although osteoarthritis (OA) is a multifactorial disease, little has been reported regarding the cooperative interaction among these factors on cartilage metabolism. Here we examined the synergistic effect of ovariectomy (OVX) and excessive mechanical stress (forced running) on articular cartilage homeostasis in a mouse model resembling a human postmenopausal condition.

**Methods:**

Mice were randomly divided into four groups, I: Sham, II: OVX, III: Sham and forced running (60 km in 6 weeks), and IV: OVX and forced running. Histological and immunohistochemical analyses were performed to evaluate the degeneration of articular cartilage and synovitis in the knee joint. Morphological changes of subchondral bone were analyzed by micro-CT.

**Results:**

Micro-CT analyses showed significant loss of metaphyseal trabecular bone volume/tissue volume (BV/TV) after OVX as described previously. Forced running increased the trabecular BV/TV in all mice. In the epiphyseal region, no visible alteration in bone morphology or osteophyte formation was observed in any of the four groups. Histological analysis revealed that OVX or forced running respectively had subtle effects on cartilage degeneration. However, the combination of OVX and forced running synergistically enhanced synovitis and articular cartilage degeneration. Although morphological changes in chondrocytes were observed during OA initiation, no signs of bone marrow edema were observed in any of the four experimental groups.

**Conclusion:**

We report the coordinate and synergistic effects of extensive treadmill exercise and ovariectomy on articular cartilage degeneration. Since no surgical procedure was performed on the knee joint directly in this model, this model is useful in addressing the molecular pathogenesis of naturally occurring OA.

## Background

Osteoarthritis (OA) is a group of diseases and mechanical abnormalities involving degradation of articular cartilage and subchondral bone. Since OA is considered a multifactorial disease, the molecular pathogenesis of OA has proven difficult to fully elucidate [1]. Epidemiological analyses have identified several factors significantly associated with OA prevalence. These include joint injury [2], joint instability (abnormal and/or excessive mechanical stress) [3, 4], joint inflammation [5, 6], menopause [7, 8], gender, aging, race [9, 10], and obesity [11, 12]. Recent studies have revealed evidence that these risk factors may intricately and cooperatively contribute to both the initiation and progression of OA. Several prospective studies have confirmed that high body mass index (BMI) affects disease incidence more than it affects disease progression [13–15]. This may suggest that the combination of obesity and other mechanical factors, such as joint alignment and extensive stress, initiate OA. Other reports have suggested that obesity and estrogen have implications in the process of disease development as the association between high BMI and knee OA appears to be stronger in women compared with men [9–12]. Inflammation inside and in the surrounding region of the joint, including synovitis and bone marrow lesions (BML), is considered to be a major factor associated with the risk of both the progression and symptoms of OA such as joint pain [16–18]. The inflammation of the synovial membrane is reported to occur in both the early and late phases of OA and is associated with alterations in the adjacent cartilage tissues [19]. Synoviocytes are metabolically highly active cells residing in the synovial membrane and are considered physiologically prerequisite for joint homeostasis as they nourish chondrocytes via synovial fluid and remove metabolites and matrix degradation products from joint space. However, in an inflammatory environment, it is reported that these cells express catabolic and proinflammatory mediators such as cytokines, nitric oxide, prostaglandin E2, and neuropeptides, which leads to excess production of the proteolytic enzymes responsible for cartilage degeneration [16]. These evidences suggest the relevance of obesity, inflammation, and estrogen signal in disease pathogenesis.

Both prevention of cartilage loss and promotion of cartilage repair in the recovery of joint function are important issues to address for both the aged and young population. However, no etiotropic treatment for OA has been developed to date, and the current major therapeutic strategy for OA is based on conservative treatments such as muscle exercise with medication to relieve joint inflammation and pain [20]. One reason for this may be the complexity of the pathogenesis of OA as described above. To address the molecular pathogenesis of OA, several experimental OA models have been established using laboratory animals. These include surgical OA models involving conditions such as destabilization of the medial meniscus (DMM) [21], anterior cruciate ligament transection (ACL-T) [22], and menisectomy [3]. Other models use toxins or small chemical compounds which induce chondrocyte apoptosis or inflammation in the joint. Monosodium iodoacetate (MIA) or carrageenan are commonly used in these models [23, 24]. However, all these models seem to involve the pathogenesis of secondary (traumatic) OA, which represents less than 15 % of total OA in humans [25].

This study was aimed to establish a new mouse OA model, which resembles the initiation and progression of human idiopathic (primary) OA. For this purpose, we examined the effect of extensive treadmill exercise (excessive mechanical stress) and ovariectomy (OVX) on articular cartilage homeostasis, both of which have been indicated as risk factors for human OA. We report both the coordinate and the synergistic effects of extensive treadmill exercise and ovariectomy on articular cartilage degeneration. Since no artificial procedure was performed on the knee joint directly in this model, we believe that this model is useful in addressing the molecular pathogenesis of idiopathic OA.

## Methods

### Animal protocol

**Fig. 1:**
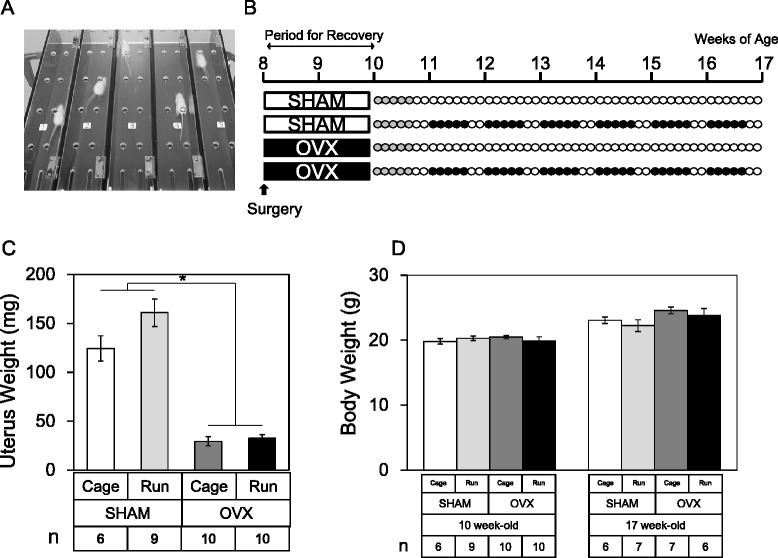
Study design. **a** A picture of the treadmill apparatus used in this study. **b** Schematic overview of the experimental protocol. Female Balb/cCrSlc mice were randomly divided into four groups. At 8 weeks of age, mice underwent OVX or SHAM surgery with 2 weeks for recovery. At 10 weeks of age, mice were subjected to a training period of forced running at 12 m/s for 10 min as an adaptation to treadmill running (5 days a week, gray circle). After the initial training period, “Run” groups were then subjected to forced running at 12 m/s for 10 min followed by 20 m/s for 100 min for 6 weeks (*closed circle*) and “Cage” groups were left in cage *ad libitum* (*open circle*). After 6 weeks of running or cage *ad libitum*, the uterus and both left and right knee joints were harvested. **c** Uterus weights were significantly decreased after OVX regardless of running. Values are means with SEM. Mann-Whitney’s U test was performed between the SHAM group (*n* = 15) and the OVX group (*n* = 20). *Asterisk* indicates *p* = 0.0002. **d** Body weights before and at sacrifice. Values are means with SEM. Differences among each group were assessed using a Kruskal-Wallis one-way analysis of variance by ranks followed by Steel -Dwass’s post-hoc test. Removal of ovary did not alter body weight significantly at 2 weeks after surgery (“10 week-old”, *P* = 0.59) and after forced running between the groups (“17 week-old”, *P* = 0.12). Number of mice in each group is indicated in the figure

Animal protocols were approved by the Institutional Animal Care and Use Committee (IACUC) of Tokyo Medical and Dental University (#0140079A). Eight week-old female Balb/cCrSlc mice were randomly divided into two groups: the OVX group (OVX) and the control group (SHAM). Two weeks after surgery, all mice were subjected to forced running for 5 days at 12 m/min for 10 min followed by 20 m/min for 10 min using a rodent treadmill machine (Fig. 1a, MK-680R5; ME Service, Tokyo, Japan). Each group was further divided into two groups: one group was subjected to forced running by treadmill (OVX + Run or SHAM + Run) and the other group was left in the cage *ad libitum* (OVX + Cage or SHAM + Cage). The running group was forced to run 60 km for an additional 6 weeks (5 days per week) at 12 m/min for 10 min of warm-up followed by 20 m/min for 100 min (Fig. 1b). The number of mice in each experimental group is indicated in each figure. Mice were housed under a 12-h light-dark cycle and allowed food and water *ad libitum*. After 6 weeks, all the mice were sacrificed, and the uterus was extracted and weighed. Both left and right knee joints were also harvested for histological analyses. In addition, body weight was recorded before and after the running protocol. The humane endpoint was defined in this experiment according to the IACUC guidelines. If we observed indications of non-specific signs of illness such as inactivity, hunched posture or a rough coat during forced running by treadmill, experiments were terminated and mice were euthanized by carbon dioxide. Frequency of dropouts during treadmill running was about 30 % in this experiment.

### Macroscopic observation of articular cartilage

Right femurs were carefully dissected and articular cartilage was stained using India ink to visualize the damaged region as described by Sekiya et al. [4].

### Histology

Left knee joints were harvested, and placed on a rubber plate at fixed knee joint angles (30°), fixed in 4 % PFA (paraformaldehyde, SIGMA Aldrich, MO), demineralized in 20 % EDTA (ethylenediaminetetraacetic acid) and embedded in paraffin. Five micrometer serial sagittal sections were prepared in the medial joint compartment, 150 +/−20 μm from the anterior cruciate ligament attachment site, for histological and immunohistochemical analyses. To evaluate cell infiltration and hyperplasia of the synovium, hematoxylin and eosin (H&E) staining was performed. The severity of synovitis was evaluated as described by Yamada et al. (see below) [24]. To examine the loss of proteoglycan from articular cartilage, safranin-O staining was performed as we previously described [4]. The severity of articular cartilage damage was evaluated by the modified Mankin’s score (see below) [2].

### Morphological analysis of articular cartilage

Morphological parameters were evaluated as described by Abula et al. [26]. To evaluate chondrocyte cellularity, the size of each chondrocyte lacunae, and chondrocyte density in the articular cartilage, an area of articular cartilage was selected between the anterior and posterior edges of the meniscus. Carl Zeiss Axiovision software (Jena, Germany) was employed for the evaluation. Chondrocytes were counted and divided by the articular cartilage area to obtain chondrocyte density.

### Immunohistochemistry

Paraffin-embedded sections were de-paraffinized in xylene, rehydrated through graded alcohol, and immersed in PBS. The samples were pretreated with 0.4 mg/ml proteinase K (DAKO, Carpinteria, CA, USA) in a Tris-HCl (pH 7.6) buffer for 15 min at room temperature for antigen retrieval. Any residual enzymatic activity was removed by washing with PBS, and nonspecific staining was blocked by pre-incubation with PBS containing 10 % normal horse serum for 20 min at room temperature. Rabbit monoclonal anti-collagen type Ia1 and anti-collagen type IIa1 were purchased from Abcam (ab34710 and ab34712 Cambridge, MA, USA). Rat monoclonal anti-F4/80 was purchased from Bio-Rad (MCA497R Hercules, CA). Anti-collagen type I, type II collagen, and F4/80 (type I collagen 1:200 dilution; type II collagen 1:1000 dilution; F4/80 1:2000) were placed on each section for one hour at room temperature. After extensive washing with PBS, the sections were incubated in the secondary antibody of biotinylated anti-rabbit and rat IgG (Vector Laboratories, Burlingame, CA, USA) for 30 min at room temperature. Immunostaining was detected with VECTASTAIN ABC reagent (Vector Laboratories), followed by DAB staining. Counter staining was performed with Mayer’s hematoxylin.

### Bone morphometric analysis (Micro computed tomography)

To analyze the structural alteration of the knee joint after treadmill running and/or OVX, right femurs were harvested and fixed in 70 % ethanol. Skeletal imaging was captured using a micro-CT apparatus (Scan Xmate-E090; Comscan Techno Co., Kanagawa, Japan). Both metaphyseal and epiphyseal bone volume per tissue volume (BV/TV), trabecular spacing (Tb. Spac.), trabecular thickness (Tb. Th.), trabecular number (Tb. N.), and subchondral bone plate thickness (Sb. Pl. Th.) of the tibia were calculated using Tri/3D-BONE software (Ratoc System Engineering Co., Tokyo, Japan). The trabecular bone parameter was measured in the metaphyseal region 300–800 μm proximal from the edge of the growth plate.

### Modified Mankin’s score

Articular cartilage structure, tidemark duplication, safranin-O staining, fibrocartilage, chondrocyte clones in uncalcified cartilage, and hypertrophic chondrocyte in calcified cartilage and subchondral bone were scored in both the femur and the tibia according to the modified Mankin’s score (summed for a maximum score of 60) [2].

### Synovitis score

Synovitis score was calculated as previously described by Yamada et al. [24]. Briefly, H&E slides were used to evaluate synovial activation by scoring the thickening of the synovial lining and influx of inflammatory cells. In each knee joint, synovial activation was scored as follows: 0, no changes compared to normal joints; 1, thickening of the synovial lining and some influx of inflammatory cells; 2, thickening of the synovial lining and intermediate influx of inflammatory cells; and 3, profound thickening of the synovial lining (more than four cell layers) and maximal observed influx of inflammatory cells. The upper and lower sides of the medial and lateral meniscus were scored and summed for a maximum score of 12.

### Statistical analysis

Statistical analysis was performed using Mann Whitney’s U-test or Kruskal-Wallis one-way analysis of variance by ranks followed by Steel-Dwass’s post-hoc methods. *P* < 0.05 was considered as significant. Data is presented as the mean +/− standard error of the mean (SEM).

## Results

### OVX and forced running synergistically accelerates articular cartilage degeneration in the knee joint

**Fig. 2:**
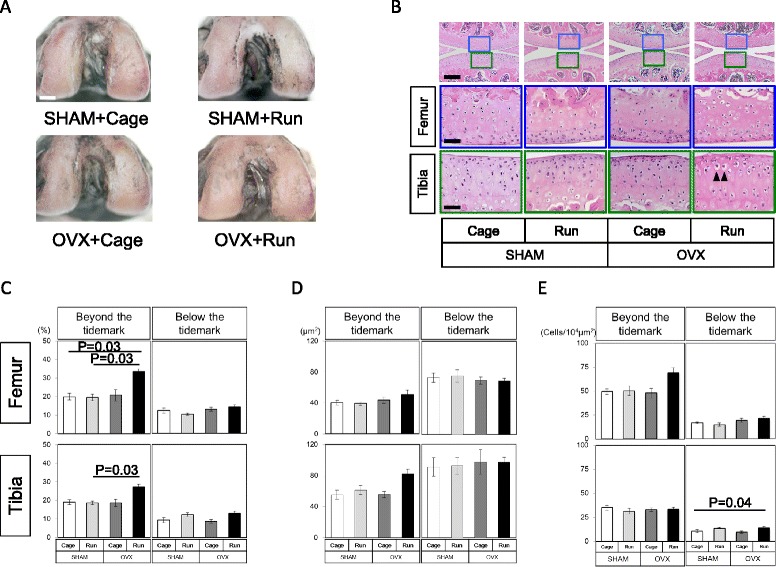
Macroscopic features and morphological analysis of articular cartilage. **a** Macroscopic observation of femoral articular cartilage stained with India ink. Typical images of the femoral cartilage surface from each of the four experimental groups. In all groups, cartilage surface seemed to remain smooth and similar India ink dyeability was observed macroscopically. Scale bar: 1 mm. Number of mice is 6 in each group. **b** Histological observation from each group stained with H&E (*n* = 6 in each group). *Boxed* areas in the top panels are magnified and presented in the bottom panels. Scale bar: top 100 μm, middle and bottom 25 μm. *Arrowheads* in OVX + Run group indicate hypertrophic differentiation of articular chondrocytes. **c** The chondrocyte cellularity of articular cartilage (%), **d** the size of each chondrocyte lacunae (μm^2^), and **e** chondrocyte density (cells/10^4^ μm^2^) of the femur and the tibia between groups. Values are means with SEM. Differences among each group were assessed using a Kruskal-Wallis one-way analysis of variance by ranks followed by Steel-Dwass’s post-hoc test. An *asterisk* indicates a statistically significant difference between the groups (*n* = 6)

Weights of the uterus in the OVX group were significantly lower compared to those in the SHAM groups, indicating the complete removal of the ovary in the OVX group (Fig. 1c, *p* = 0.0002). Since body weight is considered to be an important factor in articular cartilage homeostasis [12], we compared the average disparity of body weights between the groups during the experiment. As shown in Fig. 1d, regardless of OVX and forced running, we observed quite comparable body weights between the groups throughout the experimental period. In addition, macroscopic observation indicated the gross appearance of articular cartilage was quite comparable between the experimental groups. As shown in Fig. 2a, the cartilage surface remained smooth and we observed similar India ink dyeability regardless of OVX and forced running. However, we observed significant morphological changes in articular chondrocytes in the OVX group after forced running. As shown in Fig. 2b, ectopic hypertrophic chondrocytes were observed in the superficial zone of the tibia in the OVX + Run group (Fig. 2b arrow heads).

To further analyze articular cartilage morphology quantitatively, we measured cellularity (% area of chondrocyte lacunae, Fig. 2c), size of chondrocyte (μm^2^, Fig. 2d), and chondrocyte density (cells/10^4^ μm^2^, Fig. 2e) both beyond and below the tidemark of articular cartilage. As shown in Fig. 2c, cellularity in the superficial zones of articular cartilage was significantly increased in the OVX + Run group beyond the tidemark (*p* = 0.03). In the tibia, we found that the average size of each articular chondrocyte lacuna was increased beyond the tidemark in the OVX + Run group, although it was not statistically significant (*p* = 0.06). Quantitative analyses also indicated that the number of the cells was increased in the superficial layer of femur of the OVX + Run group (upper left panel, Fig. 2e, *p* = 0.06) and in the deep layer of tibia (lower right panel, Fig. 2e, *p* = 0.04).

**Fig. 3:**
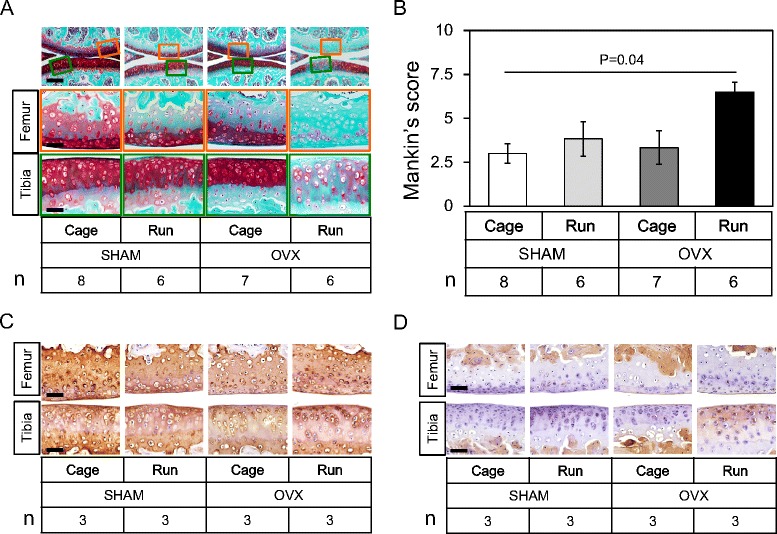
OVX and forced running synergistically accelerates articular cartilage degeneration in the knee joint. **a** Histological observation from each group stained with safranin-O. *Boxed* areas in the top panels are magnified and presented in the bottom panels. We observed significant loss of safranin-O dyeability in the OVX + Run group. Scale bar: top 100 μm, middle and bottom 25 μm. Number of mice in each group is indicated in the figure. **b** Mankin’s score for the femoral and tibial compartment between groups. The score confirmed that OVX and forced running synergistically accelerated articular cartilage degeneration. Values are means with SEM. Differences among groups were assessed by a Kruskal-Wallis one-way analysis of variance by ranks followed by Steel-Dwass’s post-hoc test. Number of mice in each group is indicated in the figure. **c**, **d** Immunohistochemistry for type II (**c**) and type I collagen (**d**) in each group. Ectopic expression of type I collagen in both femoral and tibial articular cartilage was observed in the OVX + Run group. Scale bar: 25 μm. Number of mice in each group is indicated in the figure

To assess the effect of OVX and forced running on cartilage matrix degeneration, histological and immunohistochemical analyses were performed. As shown in Fig. 3a, OVX and forced running respectively had subtle effects on safranin-O dyeability of articular cartilage. However, we observed significant loss of safranin-O dyeability in the OVX + Run group (Fig. 3a the rightmost column). To semi-quantitatively evaluate articular cartilage degeneration, the modified Mankin’s score was calculated and plotted in Fig. 3b. The modified Mankin’s score confirmed that OVX and forced running synergistically accelerated articular cartilage degeneration (*P* = 0.04). Immunohistochemical analyses indicated comparable levels of type II collagen expression in both femoral and tibial articular cartilage regardless of OVX and forced running (Fig. 3c). In contrast, we observed ectopic expression of type I collagen in both femoral and tibial articular cartilage in the OVX + Run group (Fig. 3d).

### Effect of OVX and forced running on bone morphology

**Fig. 4:**
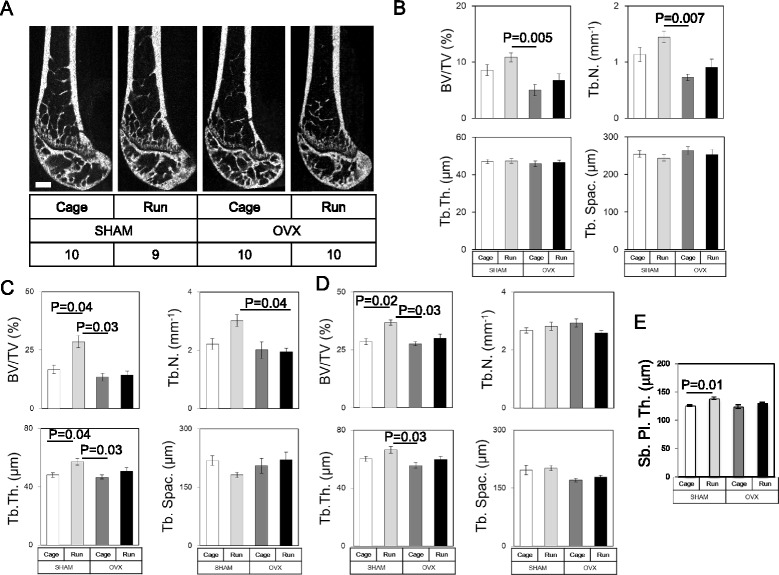
Changes in bone structure and the subchondral trabecular bone in each group. **a** Representative μCT images. No apparent osteophyte formation or bone deformation around the knee joint was observed by μCT in any of the groups. Scale bar: 500 μm. Number of mice in each group is indicated in the figure. **b** Trabecular bone volume fraction (BV/TV), trabecular number (Tb. N.), trabecular thickness (Tb. Th.), and trabecular spacing (Tb. Spac.) of the metaphysis of the femur. Forced running tended to increase BV/TV and Tb.N. and OVX completely abolished these anabolic effects. **c** Bone morphometrical analyses of the metaphyseal region of tibia. Similar results were observed if compared to those in femur. **d** Bone morphometrical analyses of the epiphyseal region of tibia. Forced running increased BV/TV and OVX completely abolished this anabolic effect. **e** Subchondral bone plate thickness (Sb. Pl. Th.) of the tibia was shown. Forced running increased Sb. Pl. Th. and OVX completely abolished this anabolic effect. Values are means with SEM. Differences among each group were assessed using a Kruskal-Wallis one-way analysis of variance by ranks followed by Steel-Dwass’s post-hoc test. *P* values are indicated if the difference was statistically significant. Number of mice in each group, **b**: SHAM + Cage = 10, SHAM + Run = 9, OVX + Cage = 10, and OVX + Run = 10, **c**-**e**: SHAM + Cage = 8, SHAM + Run = 6, OVX + Cage = 6, and OVX + Run = 6

Since abnormal subchondral bone metabolism such as osteoporosis, sclerosis, as well as osteophyte formation in the joint are thought to be associated with articular cartilage degeneration [27, 28], we performed bone morphometric analyses after OVX and forced running (Fig. 4). No apparent osteophyte formation or bone deformation around the knee joint was observed by micro-CT in any of the groups (Fig. 4a). Micro-CT analyses revealed that forced running increased both BV/TV and Tb. N. in the metaphyseal region of femur regardless of OVX, although these were not statistically significant (compare Cage and Run regardless of OVX, Fig. 4a and b). OVX reduced BV/TV (compare SHAM and OVX regardless of running, Fig. 4a and b). In OVX mice, forced running for 6 weeks partially reversed BV/TV and Tb.N. up to the control level (SHAM + Cage) although this difference was not statistically significant (compare OVX + Cage and OVX + Run, Fig. 4b). In contrast, we did not observe any significant alteration in Tb.Th. and Tb. Spac. between the groups (Fig. 4b).

We observed distinct results in the metaphyseal (Fig. 4c) and epiphyseal (Fig. 4d) region of the tibia. BV/TV was significantly increased after forced running in both metaphysis (*p* = 0.04) and epiphysis (*p* = 0.02) in control mice (compare SHAM + Cage and SHAM + Run in Fig. 4c, d). Interestingly, OVX abolished this effect (compare OVX + Cage and OVX + Run in Fig. 4c, d). In stark contrast to BV/TV, Tb. N. was not increased in both metaphyseal and epiphyseal region of the tibia. Metaphyseal Tb. Th. was significantly increased by forced running in sham mice (compare SHAM + Cage and SHAM + Run in Fig. 4c, *p* = 0.04). Similar to BV/TV, this effect was also deteriorated by OVX. We did not observe any significant alteration in Tb. Spac. between the groups (Fig. 4c and d). Thickness of the tibial subchondral bone plate was significantly increased by forced running (Fig. 4e, *p* = 0.01), which was also deteriorated by OVX.

### OVX and forced running coordinately enhance joint inflammation

**Fig. 5:**
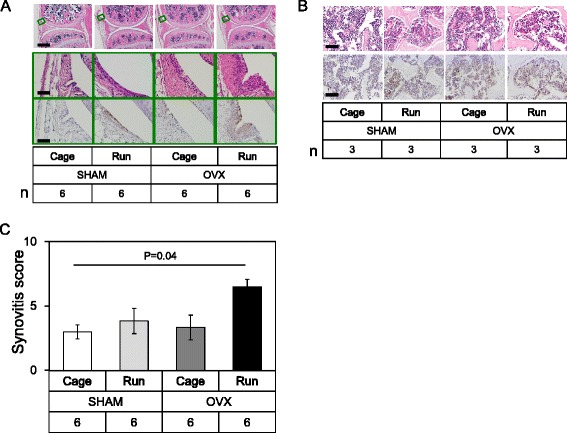
Inflammatory response in each group. **a** H&E staining and immunohistochemistry for F4/80 in the joint. *Boxed* areas in the top panels are magnified and presented in the bottom panels. *Top and middle panels*; H&E staining, bottom panel; immunohistochemistry for F4/80 in the synovial membrane. Scale bar: top 250 μm, middle and bottom 25 μm. Number of mice in each group is indicated in the figure. **b** Histochemical analysis of epiphyseal bone marrow cavity of the femur. Scale bar: 25 μm. Top panels, H&E staining. *Bottom panels*, F4/80 staining. Number of mice in each group is indicated in the figure. **c** Semi-quantitative analysis of synovial inflammation. Values are means with SEM (*n* = 6). Differences among each group were assessed using a Kruskal-Wallis one-way analysis of variance by ranks followed by Steel-Dwass’s post-hoc test. *P* value is indicated if the difference is statistically significant

Since inflammatory responses, such as inflammation in the synovial membrane and bone marrow edema in the epiphyseal bone marrow cavity were frequently observed at the onset of OA in the knee joint [16–18], we next probed for signs of joint inflammation in each group. In the SHAM + Run and OVX + Cage group we observed mild synovial hyperplasia and macrophage infiltration into the synovial membrane, both of which are indicators of joint inflammation (Fig. 5a). Interestingly, OVX and forced running coordinately enhanced joint inflammation (Fig. 5a, OVX + Run, Fig. 5b). In contrast, we did not observe any significant change in cellularity or macrophage accumulation in the epiphyseal bone marrow cavity of the femur between the groups.

## Discussion

In this study, we established a new mouse model which resembles naturally occurring OA phenotypes by combination of OVX and extensive mechanical stress to the knee joint. In this model, signs of early OA including ectopic type I collagen deposition, hypertrophic differentiation of articular chondrocytes, significant loss of proteoglycan, and synovitis were observed in the joint. We consider that this model is useful in addressing the molecular pathogenesis of naturally occurring OA since surgical procedure was not performed directly on the knee joint. Furthermore, this model will be useful to investigate the crosstalk between the signals activated post menopause and signals transduced by abnormal mechanical stress to the knee joint.

Menopause is a physiologic event characterized by loss of ovarian hormones and which can cause various disorders such as coronary heart disease, vasomotor symptoms, and osteoporosis [29]. In addition to mechanical stress such as joint instability [3] and extensive loading [4, 7, 8, 30, 31], menopause is a major contributor to the degeneration of articular cartilage. In the current study, OVX induced significant bone loss. However, it did not induce significant effects on cartilage or synovium. Lugo et al. and Romen-Blas et al. reported that estrogen has both direct effects on cartilage and indirect effects on subchondral bone, muscle, ligament, and synovial membrane via estrogen receptor for joint homeostasis [32, 33]. Moreover, OVX is often accompanied by an increase in fat mass [34], which leads to increased levels of adipose tissue derived cytokines such as leptin and interleukin-6 (IL-6) [35, 36], which may also contribute to joint inflammation and OA progression. Sniekers et al. demonstrated that estrogen depletion increased the susceptibility of tissues in the joint for changes. However, an additional trigger induced by iodoacetate was needed to initiate osteoarthritic changes [37]. Taken together, our findings support the theory that additional triggers are required to initiate OA in postmenopausal women.

Exercise is widely recommended as one of the key preventive strategies for OA as well as osteoporosis. There are several animal studies reporting both the anabolic and catabolic effects of exercise on articular cartilage [4, 38–40], which suggest that moderate exercise is generally protective even after the onset of OA, although excessive exercise can lead to OA initiation and progression. Mechanical loading is reported to regulate the PTHrP/Ihh axis and presumed to be chondroprotective [41]. In our study, cartilage degeneration was always associated with excessive conditions, although the effect was not statistically significant when compared to control mice. This suggests that differences in age, strain, and species might also contribute to the susceptibility of OA initiation and progression.

Since OA is considered to be a degenerative joint disease characterized by cartilage breakdown as a consequence of wear and tear [42], the synovial membrane, and its inflammation have also been reported to play a physiologically important role on joint homeostasis and cartilage degradation as reported by Sellam et al. [16]. In addition, Blom et al. reported that synovial macrophages were crucial to cartilage degeneration in mediating Matrix metalloproteinase (MMP) activity in synovial macrophage knockout mice [43]. Several studies have indicated that inflammatory responses, such as bone marrow edema in the epiphyseal bone marrow cavity and inflammation in the synovial membrane, were frequently observed at the onset of OA in the knee joint. [16–18] As with previous reports, we observed the infiltration of macrophages into the synovial membrane in the OVX + RUN group, which might lead to the initiation of OA. Conversely, we found no alteration in bone marrow lesions between the 4 groups, although Roemer et al. reported the correlation between cartilage loss and bone marrow edema in a human study [17]. In addition, we found no apparent morphological alterations in deep zone cartilage despite significant morphological alterations in the superficial zone. Taken together, our results suggest that the synovium might account for OA initiation more than the bone marrow.

Type I collagen expression is believed to be a fibrocartilage marker [44]. Even though no decrease of type II collagen expression or alteration of the joint surface was observed macroscopically, fibrosis may have already occurred followed by an inflammatory response. In addition, our results also showed that the cellularity and hypertrophic chondrocytes were increased as well as ectopic type I collagen expression on articular cartilage in the OVX + Run group. Generally, hypertrophy of chondrocytes occurs during articular cartilage degeneration [3, 45]. Our data suggest that the combination of mechanical stress and the loss of ovary function enhanced the proliferation and hypertrophic differentiation of articular chondrocytes. Meanwhile, morphological changes in deep zone cartilage were not observed in any of the groups. One reason for this may be the fact that our current model represented a relatively early stage of OA compared to previous studies [17, 46]. In addition, we believe that the increased chondrocyte cellularity, particularly in the upper cartilage zones, may be due to the fact that superficial cartilage becomes more exposed to factors from the synovium compared to deep zone cartilage.

Botter et al. reported that porosity in subchondral lesions due to increased osteoclastic activity was present in mouse OA induced by collagenaseVII [47]. As shown in Fig. 4, our study revealed that treadmill running always improved bone mineral parameters such as BV/TV and Tb.Th of both the femur and the tibia in accordance with previous human and animal studies [48, 49]. However in most case, OVX completely abolished these anabolic effects. These data suggest the abnormal mechanotransduction in bone cells in the absence of estrogen signal. This may be one reason why we observed accelerated degradation in articular cartilage in OVX + Run group, further mechanistic analyses are required to elucidate this issue.

There are several limitations to the current study. First, although we reported the bone parameter of both the epiphysis and the metaphysis of the tibia, we could not examine the epiphysis of the femur according to the geometries of the epiphysis. However, the fact that the metaphysis bone parameter of the femur was similar to that of the tibia may suggest a similar correlation between the femur and the tibia on joint homeostasis. Second, the surgical approach was applied to young adult mice, and did not fully simulate the natural conditions of elderly postmenopausal women. Third, we focused on the synergistic effect of OVX and excessive mechanical stress, but we did not include estrogen supplementation in this study for the following reasons. Several studies have investigated the relationship between OVX and estrogen supplementation in young animals, lacking the premise that OVX is an established procedure which stimulates the hormonal condition of postmenopausal women; however, OVX not only changes estrogen levels, but also levels of progesterone (down), follicle-stimulating hormone (up), luteinizing hormone (up), and testosterone (down). Therefore, in surgical menopause models it is possible that estrogen supplementation might not compensate OVX. This may also explain why subsequent follow up analysis failed to show significant estrogen replacement therapy (ERT) protection against either the development or progression of radiographic knee OA in previous studies [50, 51]. Fourth, we did not observe the long term combined effect of OVX and forced running. Since immunohistochemistry for type II collagen showed no apparent alteration in any of the four groups, a longer follow up period is necessary to assess any further alteration in type II collagen and its potential effect on OA change.

## Conclusions

We established a new mouse model which resembles naturally occurring OA phenotypes by combination of OVX and extensive mechanical stress to the knee joint. We consider that this model is useful in addressing the molecular pathogenesis of naturally occurring OA since surgical procedure was not performed directly on the knee joint. Furthermore, this model will be useful to investigate the crosstalk between the signals activated post menopause and signals transduced by abnormal mechanical stress to the knee joint.

## Abbreviations

OA, osteoarthritis; OVX, ovariectomy; BV/TV, bone volume/tissue volume; Tb.Th., trabecular thickness; Tb.N., trabecular number; Tb.Spac., trabecular space (separation); Sb.Pl.Th., subchondral bone plate thickness.
